# PPARδ status and mismatch repair mediated neoplasia in the mouse intestine

**DOI:** 10.1186/1471-2407-6-113

**Published:** 2006-05-03

**Authors:** Karen R Reed, Owen J Sansom, Anthony J Hayes, Andreas J Gescher, Jeffrey M Peters, Alan R Clarke

**Affiliations:** 1 Cardiff School of Biosciences, Cardiff University, Museum Avenue, Cardiff CF103US, UK; 2 Beatson Institute of Cancer Research, Garscube Estate, Switchback Road. Glasgow G611BD, UK; 3 Department of Cancer Studies, University of Leicester, Leicester LE2 7LX, UK; 4 Centre for Molecular Toxicology, Department of Veterinary and Biomedical Science, 312 Life Sciences Building, The Pennsylvania State University, University Park, PA 16802, USA

## Abstract

**Background:**

Therapeutic regulation of PPARδ activity using selective agonists has been proposed for various disorders. However, the consequences of altered peroxisome proliferator-activated receptor delta (PPARδ) activity in the context of intestinal tumourigenesis remain somewhat unclear. Contradictory evidence suggesting PPARδ either attenuates or potentiates intestinal neoplasia. To further investigate the PPARδ dependency of intestinal tumourigenesis, we have analysed the consequences of PPARδ deficiency upon intestinal neoplasia occurring in mice with impaired mismatch DNA repair.

**Methods:**

Mice deficient for both PPARδ and the mismatch repair gene Mlh1 were produced and the incidence and severity of intestinal neoplasia recorded.

**Results:**

No significant differences between the control genotypes and the double mutant genotypes were recorded indicating that deficiency of PPARδ does not modify impaired mismatch repair induced neoplasia.

**Conclusion:**

In contrast with the previously observed acceleration of intestinal neoplasia in the context of the *Apc*^*Min*/+ ^mouse, PPARδ deficiency does not alter the phenotype of mismatch repair deficiency. This data supports the notion that PPARδ is not required for adenoma formation and indicate that any pro-tumourigenic effect of PPARδ inactivation may be highly context dependent.

## Background

Peroxisome proliferator-activated receptors (PPARs) are lipid-activated transcription factors exerting several functions in development and metabolism. There are 3 major PPAR isoforms; α,β/δ and γ and each has distinct agonist binding properties and different regulation of expression resulting in distinct distributions [1]. The roles for PPARδ appear diverse and are not fully characterised, but include the regulation of lipid uptake, metabolism, and regulation of proliferation and differentiation within many different cell types. Consequently, the therapeutic regulation of PPARδ activity using selective agonists has been proposed for many varied disorders including: lung cancer [2], experimental autoimmune encephalomyelitis [3], skin disorders such as psoriasis and cancer [4], type 2 diabetes [5], metabolic syndrome [6] and dyslipidemias [7,8].

Although a great deal of evidence exists to show that PPARδ is potentially important in intestinal tumourigenesis, it is currently unclear whether PPARδ attenuates or potentiates this condition. Several studies have shown that activation of PPARδ or increased PPARδ levels are associated with increased intestinal neoplasia in a variety of tissues [9-13]. Also, two studies have shown that PPARδ deficiency suppressed or had no role upon tumourigenesis [14,15]. Taken together these analyses suggest that PPARδ potentiates colon carcinogenesis. However, two independent approaches have recently suggested that PPARδ expression in vivo is not up-regulated in intestinal adenomas. Firstly a study of matched human tumour and normal intestinal tissues found reduced levels of PPARδ expression in the tumours [16]. Second several studies using the classical mouse model of intestinal neoplasia (the Apc^Min ^mouse), found either no change, or reduced PPARδ expression in colonic adenomas compared to normal tissues in this model [17-20], while a recent publication has shown that ligand activation of PPARδ attenuates chemically induced colon carcinogenesis [20]. Furthermore, it has been demonstrated that PPARδ deficiency does not suppress intestinal tumourigensis in *Apc*^*Min*/+ ^mice, but indeed promotes some aspects of intestinal neoplasia [17,20,21]. These data suggest that PPARδ attenuates colon carcinogenesis.

Thus, given the obvious disparity within the published literature, we have utilized another mouse neoplasia model to further investigate the role of PPARδ in intestinal tumourigenesis. Mice possessing null mutations in the mismatch repair (MMR) gene Mlh1 are prone to develop different types of neoplasia and present with lymphomas and intestinal tumours but show increased mutation in all tissues examined [22]. Likewise, germline mutations in the human MLH1 gene are involved in Hereditary non-polyposis colorectal cancer [23]. We have inter-crossed PPARδ null mice [24] to the mismatch repair defective *Mlh1 *null mice [25] and produced cohorts for the different combinations of the genotypes in order to investigate the consequences of impaired MMR induced tumourigenesis in the context of PPARδ deficiency.

## Methods

Mice were generated from sixth generation C57BL 6 backcrossed mice. All experiments were performed according to UK Home Office regulations. Inter-crossing the PPARδ null mice (*PPAR*δ^-/-^) to the mismatch repair defective Mlh1 null mice (*Mlh1*^-/-^) produced cohorts containing a minimum of 16 animals for each genotype combination of interest (*Mlh1*^-/-^*PPAR*δ^+/+^*, Mlh1*^-/-^*PPAR*δ^+/-^*, Mlh1*^-/-^*PPAR*δ^-/-^). Littermates were genotyped by PCR on DNA from tail biopsy and allowed to age and monitored for signs of intestinal tumours. Animals were harvested when they displayed overt symptoms of disease, and tumour burden was ascertained upon dissection.

## Results and discussion

**Figure 1:**
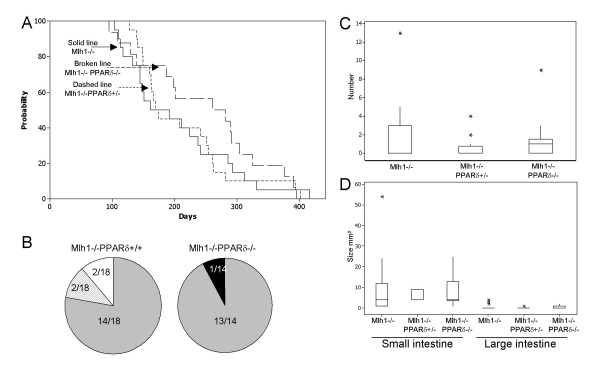
**Survival of and tumour multiplicity within each of the cohort**. Survival of (A), tumour type (B), intestinal adenoma number (C) and size (D) in *Mlh1*^-/-^*PPAR*δ^+/+^, *Mlh1*^-/-^*PPAR*δ^+/-^, and *Mlh1*^-/-^*PPAR*δ^-/- ^mice. KaplañMeier plot (A) showing the age of each animal at the time of death: Solid line, *Mlh1*^-/-^*PPAR*δ^+/+ ^(n = 20); dashed line, *Mlh1*^-/-^*PPAR*δ^+/- ^(n = 20); broken line, *Mlh1*^-/-^*PPAR*δ^-/- ^(n = 16). Pie charts (B) indicate the type identified at death in each mouse. Grey slice, mice with lymphoma; white slice, mice with akanthoma; striped slice, mice with dysplastic cysts; black slice, mice with Hemangiosarcomas. Box plots showing the number (combining small and large intestine) (C) or size (D) of intestinal adenomas per mouse at death. Intestinal preparations were collected from animals, fixed and tumours in the small and large intestine were counted and sized (expressed as width by length). Boxes encompass the first quartile (at bottom) to the third quartile (at top) of the data set; the horizontal boxed line represents the median; the asterisks represent outliers.

Through inter-crossing the PPARδ null mice (*PPAR*δ^-/-^) to the mismatch repair defective Mlh1 null mice (*Mlh1*^-/-^) we produced cohorts containing a minimum of 16 animals for each genotype combination of interest and examined survival, intestinal adenoma multiplicity and tumour size at death for each of the cohorts (Figure 1). We find that, although the mean age at death of *Mlh1*^-/-^*PPAR*δ^-/- ^mice was 248.1 days compared to 203.5 days in controls, this was not statistically different (Figure 1a, p = 0.34 Log-Rank test). Furthermore, the predisposition to lymphomagenesis was not significantly altered between the *Mlh1*^-/-^*PPAR*δ^+/+ ^and *Mlh1*^-/-^*PPAR*δ^-/- ^cohorts (Figure 1b). The possibility remains that there may be subtle effects of PPARδ deficiency that lie below the detection threshold of the present study, a possibility that would be resolved by a substantially increased cohort analysis.

Contrary to the finding from the APC^Min/+ ^*PPAR*δ^-/- ^intercross, which indicated that PPARδ deficiency promotes some aspects of intestinal neoplasia [17,20,21], no significant differences were discovered between either number or size of intestinal adenomas in the *Mlh1*^-/-^*PPAR*δ^+/+ ^and *Mlh1*^-/-^*PPAR*δ^-/- ^cohorts (Figure 1c, d). This was confirmed in both the small intestinal and large intestinal tumours (P > 0.1, Mann Whitney U test), although notably the group sizes in both these analyses were small.

To assess the nature of the *Mlh1*^-/-^*PPAR*δ^+/+ ^and *Mlh1*^-/-^*PPAR*δ^-/- ^aberrant intestinal tissues, β-catenin immunohisochemistry was performed and again no differences were observed between the two genotypes (data not shown). Up-regulation of β-catenin in large intestinal adenomas was observed in both genotypes. Furthermore in the small intestine, in addition to lesions displaying increased β-catenin levels, it was also possible to identify a small subset of lesions that maintain normal levels of β-catenin, a phenomenon previously described within defective MMR intestine [26]. Thus, the induction of dysplastic lesions and the deregulation of β-catenin in the intestine occurring as a consequence of defective MMR is not dependent upon PPARδ status and has no requirement for PPARδ.

We therefore find that loss of PPARδ does not alter tumour incidence or morphology of tumourigenesis on the *Mlh1*^-/- ^background. Given that MMR deficiency is considered to lead to a mutator phenotype which thereby increases the rate of mutation of elements of the Wnt pathway, our findings argue that PPARδ deficiency does not enhance or diminish such a mutator phenotype. By implication, the increased adenoma formation seen in the *Apc*^*Min*/+^*PPAR*δ^-/- ^mutants may arise through PPARδ dependent modification of the frequency of gene conversion events that are known to underlie the majority of polyp formation in the *Apc*^*Min*/+ ^mouse [27]. Alternatively, subsequent to the gene conversion events, PPARδ deficiency may alter the resulting cellular signalling/gene expression pathways which permit cell survival and tumour progression. Our data again apparently contradict the observed acceleration of adenoma formation following agonist activation of PPARδ[11], as this predicts reduced adenoma burden in the absence of PPARδ. However, PPARδ deficiency (as assessed through the PPARδ null mutation) may not necessarily be functionally opposite of ectopic PPARδ activation as it is possible that any alteration in the levels of PPARδ activity, whether a reduction or increase, have different consequences dependant on the genetic background or indeed tissue being studied. In terms of evaluating the potential deleterious pro-tumourigenic effect of PPARδ deficiency, our data suggests that this only accelerates adenoma formation in certain defined genetic settings (e.g. *Apc*^*Min*/+^), and does not generally enhance adenoma formation *per se*. This genetic dependency may reflect differences in the mutational events occurring in the Mlh1 and Apc mutant backgrounds. By analogy, any potential danger in the therapeutic use of PPARδ agonists to activate PPARδ may critically depend upon subtle changes in the underlying genetic predisposition.

## Conclusion

In summary, we show that PPARδ deficiency does not alter either lymphomagenesis or adenoma formation in mice with defective MMR. This data again support the notion that PPARδ is not required for adenoma formation and indicate that any pro-tumourigenic effect of inactivation may be highly context dependent. Thus, in the context of a defective MMR environment, PPARδ agonism is unlikely to be pro tumourigenic.

## Competing interests

The author(s) declare that they have no competing interests.

## Authors' contributions

KRR, OJS and AJH participated in the animal studies; KRR and OJS carried out the statistical analysis and data presentation; ARC, JMP and AJG conceived the study, and participated in its design and coordination. KRR drafted the manuscript and all authors read and approved the final manuscript.

## Pre-publication history

The pre-publication history for this paper can be accessed here:
